# Automated diagnosis of heart valve degradation using novelty detection algorithms and machine learning

**DOI:** 10.1371/journal.pone.0222983

**Published:** 2019-09-26

**Authors:** Bernhard Vennemann, Dominik Obrist, Thomas Rösgen

**Affiliations:** 1 Institute of Fluid Dynamics, ETH Zürich, Zürich, Switzerland; 2 ARTORG Center for Biomedical Engineering Research, University of Bern, Bern, Switzerland; Vietnam National University, VIET NAM

## Abstract

The blood flow through the major vessels holds great diagnostic potential for the identification of cardiovascular complications and is therefore routinely assessed with current diagnostic modalities. Heart valves are subject to high hydrodynamic loads which render them prone to premature degradation. Failing native aortic valves are routinely replaced with bioprosthetic heart valves. This type of prosthesis is limited by a durability that is often less than the patient’s life expectancy. Frequent assessment of valvular function can therefore help to ensure good long-term outcomes and to plan reinterventions. In this article, we describe how unsupervised novelty detection algorithms can be used to automate the interpretation of blood flow data to improve outcomes through early detection of adverse cardiovascular events without requiring repeated check-ups in a clinical environment. The proposed method was tested in an in-vitro flow loop which allowed simulating a failing aortic valve in a laboratory setting. Aortic regurgitation of increasing severity was deliberately introduced with tube-shaped inserts, preventing complete valve closure during diastole. Blood flow recordings from a flow meter at the location of the ascending aorta were analyzed with the algorithms introduced in this article and a diagnostic index was defined that reflects the severity of valvular degradation. The results indicate that the proposed methodology offers a high sensitivity towards pathological changes of valvular function and that it is capable of automatically identifying valvular degradation. Such methods may be a step towards computer-assisted diagnostics and telemedicine that provide the clinician with novel tools to improve patient care.

## Introduction

Heart valve disease is an increasingly common pathology of the cardiovascular system and more than 800,000 patients annually are expected to require heart valve replacement by 2050 [1]. Aortic valve (AV) replacement with a bioprosthetic heart valve (BHV) is a common therapy for a failing native AV. Despite good short- and mid-term outcomes, long-term results are limited by insufficient BHV durability [2]. The durability of BHVs depends on many factors, such as age and comorbidities [3, 4], but the exact causality of BHV failure is still not fully understood preventing accurate prediction of BHV durability. This renders the management of these valves difficult and creates a need for close monitoring of valvular function. Current diagnostic modalities include Doppler echocardiography or flow-MRI. These technologies rely on the measurement of blood flow, which is an important indicator of cardiovascular health. Valvular malfunctions show blood flow characteristics that deviate from those of the healthy AV, and these characteristic blood flow patterns are therefore used for the diagnosis of heart valve degradation. The current diagnostic procedures are labor- and cost intensive, limiting the frequency at which medical exams can be performed. Automation of data acquisition (e.g. through flow sensing implants) and diagnostic tasks has the potential to improve outcomes of AV replacement therapies by early detection of adverse cardiac events while reducing costs and minimizing hospital time. Implantable medical sensors and telemedicine continue to increase in popularity because of their potential to reduce the cost of healthcare and to enable remote patient management in areas with limited access to medical care [5–7]. These technologies allow for continuous and long-term disease monitoring that is not limited by access to clinical personnel. The rapid development of data acquisition systems that utilize implantable sensors creates a need for automated analysis of medical data that is scalable to a large patient base. Artificial intelligence and machine learning are increasingly incorporated into clinical practice and computer systems today aid the clinician in tasks that were long thought only possible for human experts [8]. Several machine learning algorithms have been previously used for the detection of heart valve diseases. These range from fuzzy logic [9–11], hidden markov models [12–14] and support vector machines [15–25] to artificial neural networks [25–35], and in recent years convolutional neural networks [36–43] and recurrent neural networks [43, 44]. Some studies combined multiple classifiers to an ensemble method to increase the accuracy of the system [24, 30, 35, 40, 45, 46]. The majority of the previous studies was based on classification of heart sounds, but little effort has been made to use such methods for the analysis of temporal patterns of blood flow rate downstream of the valve using an unsupervised learning approach. Instead, most current frameworks use supervised learning algorithms that require labeled reference datasets, which can be difficult and time-consuming to obtain. The performance of such systems can suffer if the process of patient data acquisition differs from that used for the reference data, e.g. the positioning of a handheld Doppler probe, and if physiological and pathological features are insufficiently represented in the training data. In this article, we propose the use of novelty detection algorithms to automatically detect heart valve degradation based on blood flow measurements without the need for user feedback or external reference datasets.

## Materials and methods

**Fig 1:**
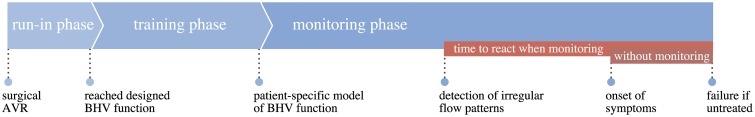
The phases of automated BHV monitoring. After aortic valve replacement (AVR), the body is given some time to adapt to the new valve. Then a physiological baseline model of the healthy BHV is trained. This model is used during the monitoring phase to automatically detect valvular degradation which may show well before the onset of clinical symptoms.

**Fig 2:**
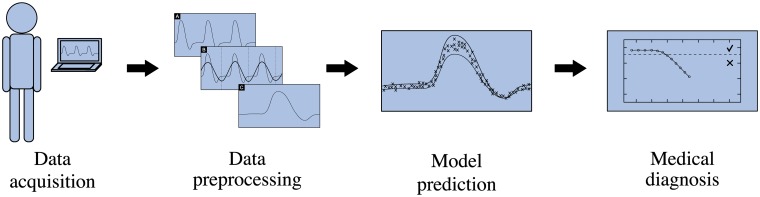
Workflow from data acquisition to automated diagnostics. The workflow includes all steps of data acquisition, data preprocessing, model prediction and automated diagnostic insight.

Our method monitors the temporal evolution of valvular function to detect progressive valve degradation. It uses training data and monitoring data that originates from the implanted valve, acquired during specific phases. These phases are illustrated in Fig 1. When a BHV is implanted, it is given some run-in time to allow for the body to adapt to the newly implanted BHV and to reach a quasi-steady state of the functioning valve. After this, the characteristic temporal patterns of blood flow rate downstream of the valve are recorded during a training phase (e.g. with an implantable flow sensor) and are used to train a patient-specific baseline model that resembles the dynamics of the healthy BHV. Other than in supervised learning approaches, this model is tailored to the implant and no information on general heart valve characteristics (e.g from an external heart valve reference database) is required. The training phase is succeeded by a monitoring phase in which blood flow recordings are acquired and are compared to the baseline model to automatically detect adverse events in form of irregular flow patterns. First indications of valvular degradation may be observed in the flow patterns well before the onset of clinical symptoms, giving more time and treatment options to restore the normal physiology [47]. Fig 2 shows the workflow during the monitoring phase from data acquisition to automated diagnostic tasks. First, blood flow data is gathered to obtain the characteristic blood flow pattern behind the AV. This data, typically spanning multiple cardiac cycles, is preprocessed to improve the signal quality and to bring it into a format suitable for computer algorithms. The data is then computer-analyzed using methods of machine learning, which yield diagnostic insight into the state of the AV. To take full advantage of the proposed method, all steps in the measurement chain should be automated as far as possible.

### Data acquisition

To enable assessment of valvular function it is necessary to measure the flow characteristics downstream of the AV. This flow data can be obtained through current diagnostic modalities, such as Doppler echocardiography or flow-MRI. Preferably, the data is acquired using a flow sensing implant, capable of in-situ flow measurement and transcutaneous data transmission. The use of such technology allows for automation of the data acquisition step, facilitating the recording of periodic measurements in a semi-continuous fashion over an extended period of time [48]. The data acquisition methodology must be capable of resolving the temporal blood flow rate profile with sufficient resolution to capture all important features of the characteristic blood flow pattern.

### Data preprocessing

**Fig 3:**
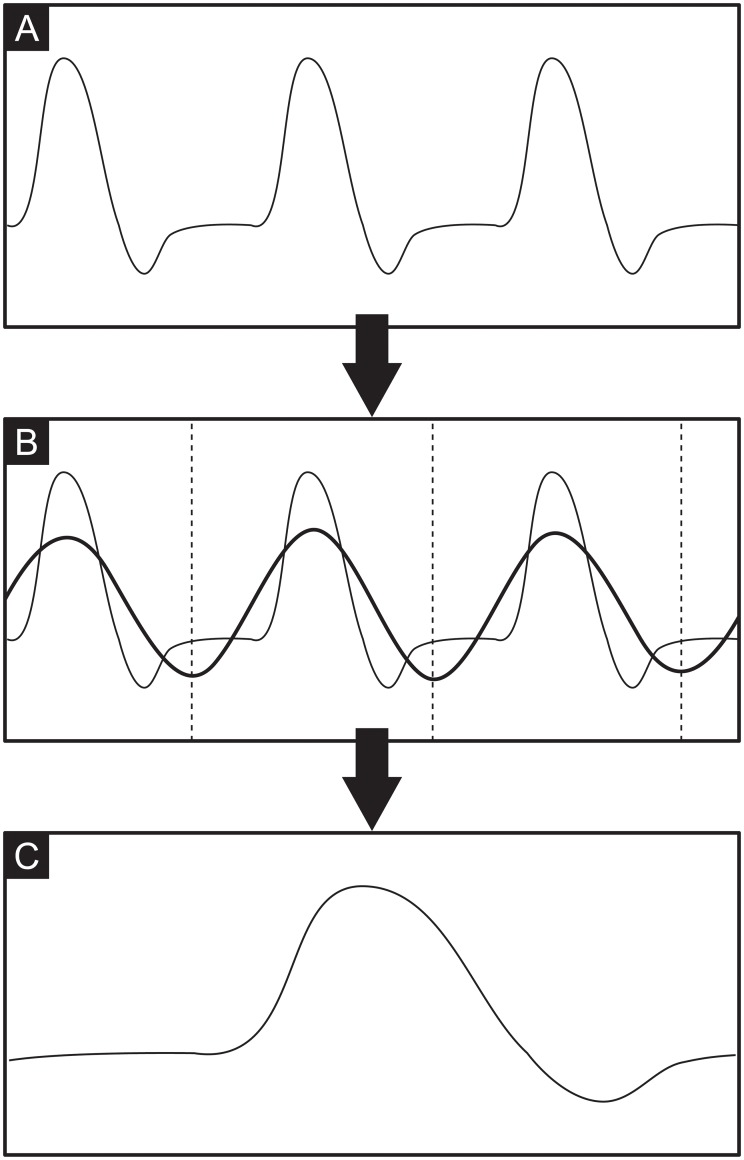
Steps of data preprocessing. A: temporal flow rate recording over multiple cardiac cycles. B: sinusoidal fit to determine phase and frequency of signal’s first harmonic. C: phase average and data standardization.

A typical flow recording contains continuously acquired data from multiple subsequent heartbeats. The preprocessing step condenses this data into a single cardiac cycle and performs feature scaling to maximize the performance of the machine learning model. This is done by fitting a sinusoid to the data, which allows identifying the instantaneous phase in the cardiac cycle from multiple heartbeats. Fig 3 shows all steps required for data preprocessing. The recording contains the instantaneous flow rate over multiple subsequent cardiac cycles (Fig 3A). A sinusoid of the form *A* sin(2*πft* + *ϕ*) + *b* is fitted to this periodic signal using a Levenberg-Marquardt algorithm [49, 50] to obtain the phase *ϕ* and the frequency *f* of the signal’s first harmonic (Fig 3B). While a sinusoidal curve fit is not a good approximation of the cardiac flow rate profile, it proved to be a very robust method to determine phase and base frequency of the signal and outperformed methods that rely on local features. The locations of the sinusoid’s extrema are then used to slice the data and to map the whole data onto a single cardiac cycle (Fig 3C) by defining a new time variable *τ* as *τ* = *t* mod *T*, where *T* is the period of one cardiac cycle. The data is subsequently scaled to have zero mean and unit variance by rescaling the original data *X* to the new variable *Z* according to

$$Z=\frac{X-\bar{X}}{\sigma },$$ (1)

where $\bar{X}$ is the arithmetic mean of *X* and *σ* is the standard deviation. This scaling, also known as standardization, brings all feature axes onto an equal scale which is a necessary precursor for optimal performance of the support vector machine (SVM) that will be used (the SVM algorithm is not scale-invariant [51]). A set of three representative features (*Z*_1_, *Z*_2_, *Z*_3_) was defined to characterize the dynamic behavior of the postvalvular flow characteristics. These are the phase in the cardiac cycle (*Z*_1_), the instantaneous flow rate (*Z*_2_) and the heart rate (*Z*_3_).

### Model prediction

**Fig 4:**
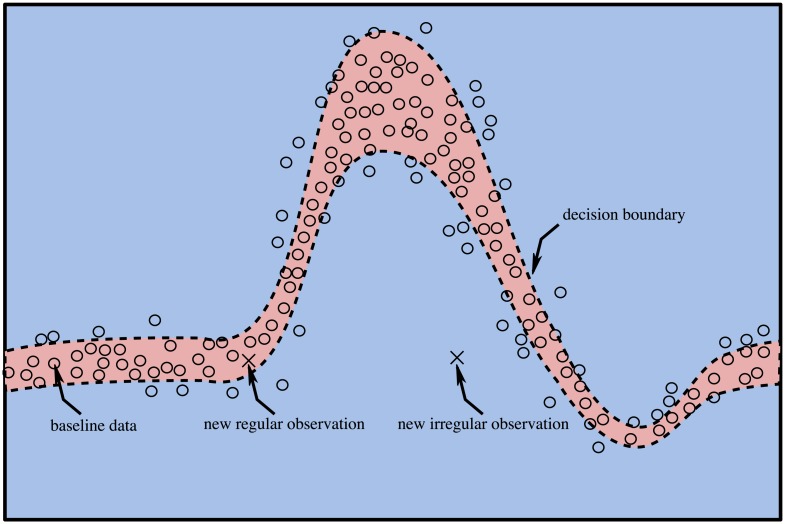
Principle of novelty detection in cardiac flow patterns. Red shaded region: inliers, blue shaded region: outliers, dashed line: decision boundary, circles: baseline data, crosses: new observation.

Flow measurements downstream of the healthy aortic valve are used to obtain a patient-specific physiological baseline. This data is recorded for the freshly implanted valve (after the body has adapted to the surgery) yielding a statistical model of the fully-operational BHV. After model training, all further measurements will be compared to this baseline model to detect valvular pathology, i.e. behavior that deviates from this baseline. We hypothesize that such a deviation can be automatically detected using a novelty detection algorithm, which is a special type in the class of anomaly detection algorithms [52], and that such a deviation from the baseline is indicative of a valvular pathology. We consider the detection of novelty sufficient for the detection of heart valve degradation despite its indifference to the type of model deviation because the evolution of valvular function over time is a unidirectional process, governed by progressive valve degradation. It is therefore sufficient to detect novelty, and not a predefined pathology because any novelty (i.e. atypical behavior) can be regarded as indicative of an underlying pathology. Obtaining a statistical model of typical behavior amounts to defining a separating hyperplane in the feature space that separates normal (physiological) states from novel (pathological) states. This hyperplane constitutes a decision boundary that is used to differentiate between normal and novel states, depending on which side of the decision boundary a given sample falls. Fig 4 illustrates the principle of novelty detection in cardiac blood flow patterns. The reference data (circles) is used to define the decision boundary that separates regular from irregular states (dashed line). A new observation that falls inside the decision boundary (red shaded region) is regarded regular, one that falls outside the decision boundary (blue shaded region) is deemed irregular (novel). In this context, a novelty corresponds to a new observation that does not originate from the same statistical distribution as the baseline model. Some of the reference data falls outside the decision boundary after model training. This situation is encountered in the presence of noise in the training data. A certain fraction of boundary violations must be permitted during model training to prevent overfitting. The result of baseline model compilation is evaluated with the corresponding model training- and test score, i.e. the agreement between model and training data and independent baseline data, respectively. It is computed by applying the baseline data to the trained classifier and by evaluating the fraction of correct classifications. The use of one-class SVMs has been successfully applied for novelty detection in datasets [53]. The one-class SVM has an important distinction from conventional SVM formulations in that it constitutes an unsupervised learning algorithm and hence does not rely on a labeled training dataset. As the name suggests, the one-class SVM consists of a single class of normal states and no further classes need to be explicitly defined during model training, in contrast to the classical SVM formulation. This property allows us to generate self-calibrating and patient-specific models of the cardiac flow characteristics that can offer a high sensitivity because the model does not need to generalize to a broad patient base, but is uniquely tailored for each subject. Schölkopf et al. [53] have formulated the mathematical description of the one-class SVM, which is briefly reviewed here.

Considering a training dataset, given by training samples ***x***_1_, …, ***x***_*l*_, it is the training objective to define a hyperplane in a high-dimensional feature space that separates this data from the origin with the largest possible margin. Finding a hyperplane that separates the data from the origin in the feature space is a concept that is special to one-class classification and builds on the principle that the origin is part of the outlier class, which allows deriving the optimal hyperplane using classical two-class SVM methodologies. The hyperplane is parameterized by a vector containing the feature weights ***ω*** and a bias term *ρ*. Maximizing the distance to the origin equates to minimizing the term ∥***ω***∥^2^. A set of slack variables *ξ*_*i*_ is introduced to prevent overfitting by allowing a predetermined fraction of boundary violations (i.e. training samples that fall onto the wrong side of the separating hyperplane after model training). This is controlled by the hyperparameter *ν* which sets the maximum fraction of allowable boundary violations and the minimum fraction of support vectors. As SVMs rely on linearly separable datasets, but real data is often highly nonlinear, the original data must be transformed into a higher-dimensional space in which the data becomes linearly separable using a transformation Φ(***x***_*i*_). The one-class SVM training objective can then be formulated as constrained optimization problem

$$\underset{\omega ,\xi ,\rho }{\text{min}}\;\frac{1}{2}{∥\omega ∥}^{2}+\frac{1}{\nu l}\sum_{i=1}^{l}\xi_{i}-\rho$$ (2)

$$\text{subject}\;\text{to}\;(\omega \cdot \Phi (x_{i}))\geq \rho -\xi_{i},\;\xi_{i}\geq 0.$$ (3)

The constraints (***ω*** ⋅ Φ(***x***_*i*_)) ≥ *ρ* − *ξ*_*i*_ and *ξ*_*i*_ ≥ 0 ensure that the training samples lie on the correct side of the hyperplane. A decision function is defined that predicts whether a given input sample should be regarded regular, or irregular and takes the form

f(x)=sgn(ω·Φ(x)-ρ). (4)

The computational complexity due to transformation of the input data into the higher-dimensional space can be greatly reduced using a kernel function of the form

$$\begin{array}{c} k(x,y)=(\Phi (x)\cdot \Phi (y)). \end{array}$$ (5)

Using suitable kernels, computations in the higher-dimensional space can be performed without actually transforming the data (“kernel-trick”) [54]. The optimization problem can be rewritten using Lagrangian multipliers *α* and the kernel definition as

$$\underset{\alpha }{\text{min}}\;\frac{1}{2}\sum_{i,j}^{l}\alpha_{i}\alpha_{j}k\left(x_{i},x_{j}\right)$$ (6)

$$\text{subject}\;\text{to}\;0\leq \alpha_{i}\leq \frac{1}{\nu l},\;\sum_{i=1}^{l}\alpha_{i}=1,$$ (7)

which yields the decision function

$$f\left(x\right)=\text{sgn}(\sum_{i=1}^{l}\alpha_{i}k\left(x_{i},x\right)-\rho ).$$ (8)

The bias term *ρ* of the decision function can be computed as

$$\rho =\left(\omega \cdot \Phi \left(x\right)\right)=\sum_{j}\alpha_{j}k\left(x_{j},x_{i}\right).$$ (9)

### Automated diagnosis

We define a diagnostic index that is indicative of the severity of valvular malfunction. It quantifies how well a given measurement agrees with the baseline model, where model agreement equates lack of novelty. This model compliance index (MCI) is defined as the fraction of measurement samples that lie within the decision boundary (inliers), compared to the total number of acquired samples of that recording,

$$\text{MCI}=\frac{N_{\text{inliers}}}{N_{\text{samples}}}.$$ (10)

This simple scalar can be monitored over time to reveal progressive valve degradation, expressed as declining MCI. A perfect agreement between measurement and baseline model would yield an MCI equal to the model training score. Deviations from the physiological baseline result in a larger fraction of samples falling outside the decision boundary and consequently a reduced MCI. Monitoring the MCI over time can yield insight into the severity and the progression rate of AV degradation.

### *In*-*vitro* test case

**Fig 5:**
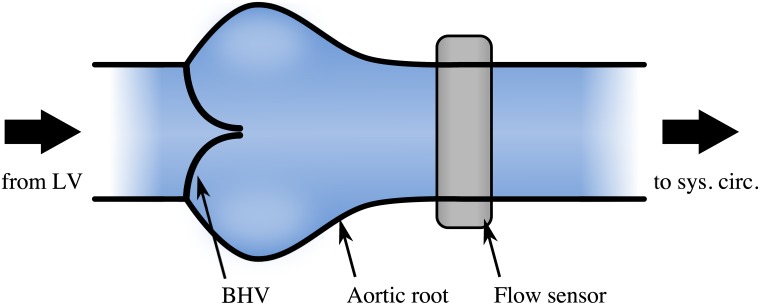
Test section including model of the aortic root with BHV and a flow sensor at the ascending aorta. A pulsatile flow loop reproduces physiological flow and pressure conditions in the aortic root. A BHV is mounted inside the aortic root and a downstream flow sensor records the temporal flow rate in the ascending aorta. LV: left ventricle.

**Fig 6:**
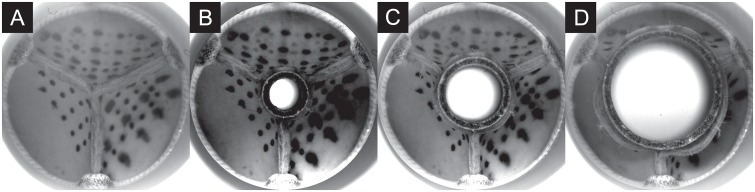
Valve during diastole with inserts creating an artificial leak. A: no AR, B: mild AR, C: moderate AR, D: severe AR.

Aortic regurgitation (AR) is a common heart valve disease in which the valve’s leaflets fail to close completely during the diastolic phase [55]. This leads to a reflux of blood from the aorta back into the ventricle. It can affect native valves and it is also a common failure mode for BHVs. We chose this common valvular pathology to test the methodology introduced above and to confirm that such a condition can be automatically detected. The scenario was imitated in an in-vitro pulsatile flow loop capable of producing flow and pressure conditions that resemble the human systemic circulation [56]. It features a test section that can host a BHV and that allows attaching a flow sensor downstream of the valve at the location of the ascending aorta (Fig 5). The system was set to physiological flow and pressure conditions for a human at rest (cardiac output 5 l/min, systolic to diastolic pressure 120/80 mmHg, heart rate 60 BPM). A magnetic flow sensor recorded the time-resolved instantaneous flow rate in the ascending aorta and was used to acquire 10 measurements of 1000 samples each at a sample rate of 60 Hz to obtain the physiological baseline [48]. The flow sensor was placed in proximity of the valve, approximately two annulus diameters downstream of the BHV to resolve the complex dynamics of the flow rate profile in the ascending aorta. The acquired data carried the characteristic signature of the valve, including important fluid-dynamic landmarks, such as peak-systolic flow, blood reflux associated with the dynamics of valve closure and any potential leakage during diastole. Artificial AR was subsequently created by placing tube-shaped inserts in the center of the valve to deliberately create AR of increasing severity by preventing full valve closure during diastole (Fig 6). A similar approach has also been used by [57]. Three differently sized inserts were used to reproduce cases of mild, moderate and severe AR, in compliance with the American Heart Association guidelines [47]. The cross-sectional area of the inserted tubes was 10.0 mm^2^, 31.4 mm^2^ and 99.4 mm^2^, respectively. All measurements were performed at a constant heart rate, which reduced the problem to two representative features, *Z*_1_ and *Z*_2_ for this setting, because the feature *Z*_3_ (heart rate) becomes obsolete.

## Results

**Fig 7:**
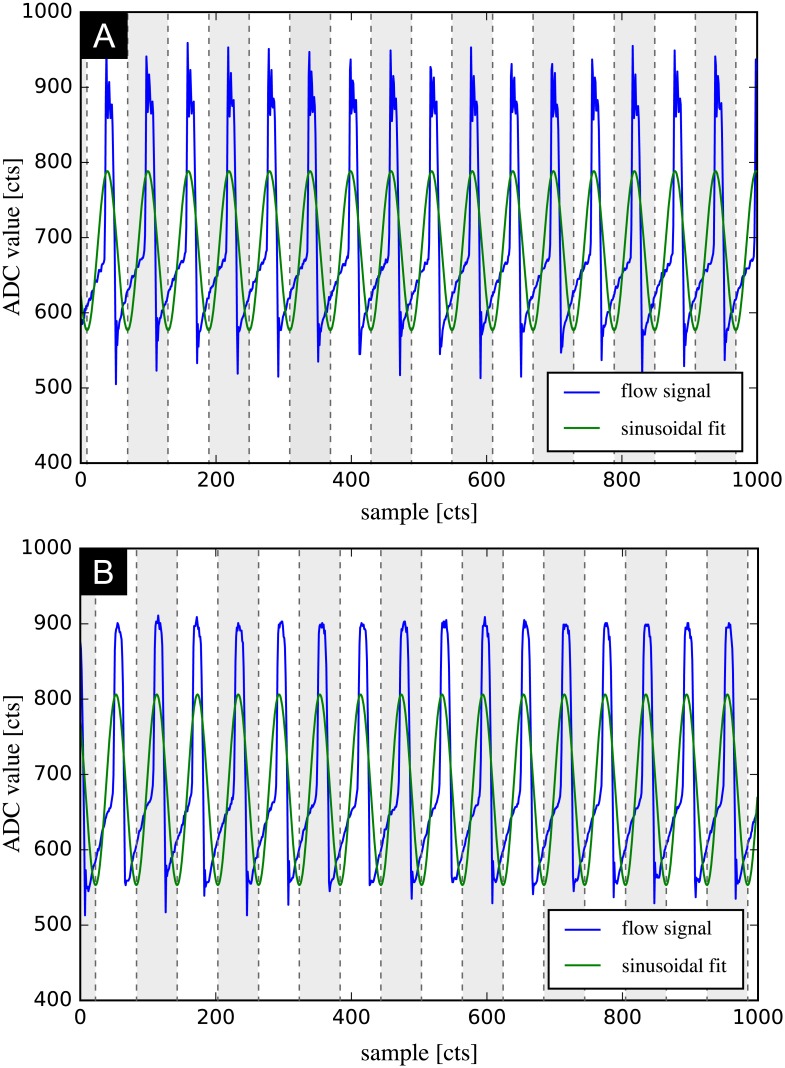
Sinusoidal fit to the measurement data. Blue line: raw data, green line: sinusoidal fit, dashed lines: slicing locations. A: baseline measurement (no AR), B: mild AR.

**Fig 8:**
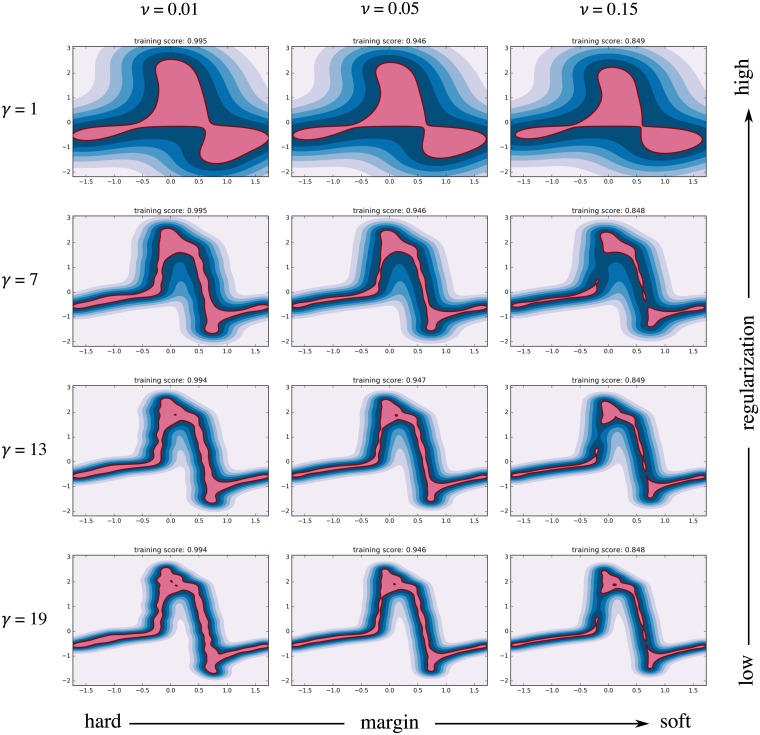
Effect of model hyperparameters on decision function. The shape of the decision function is influenced by the hyperparameters *γ* and *ν*.

**Fig 9:**
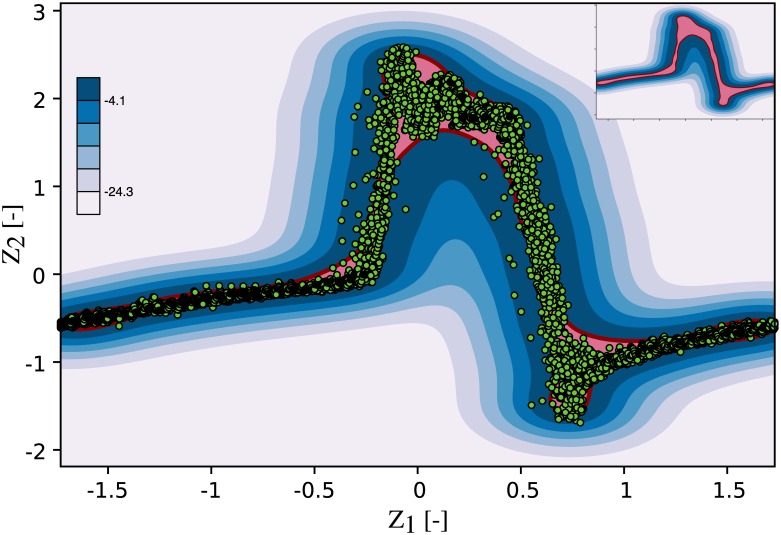
Baseline one-class SVM model. The baseline data was used to train the one-class SVM to obtain a statistical model of physiological states. Red line: decision boundary, pale red region: inlier region, shades of blue: signed distance to the decision boundary in the outlier region, green dots: baseline flow measurement.

The blue lines in Fig 7 indicate raw data as recorded by the flow sensor (A: baseline, B: mild AR), expressed as analog-to-digital converter (ADC) value. A comparison of Fig 7A and 7B illustrates that the changes in the characteristic patterns of flow rate can be subtle and easy to miss for an inexperienced observer. A sinusoid was fitted to the data (green line in Fig 7) and then sliced at the locations indicated by dashed lines. The slices were used to link and condense the data that was subsequently scaled to standardized variables. These steps were repeated for each of the baseline measurements. The preprocessed baseline measurements were then used to train the SVM to be used for novelty detection. We used the one-class SVM implementation as proposed by [53], contained in the scikit-learn package [58]. A Gaussian radial basis function (RBF) as given in Eq 11 was used as kernel function for the SVM

$$k\left(x,y\right)=e^{{-\gamma ∥x-y∥}^{2}},$$ (11)

where two hyperparameters control the shape of the resulting decision function. These are the kernel coefficient of the RBF *γ* (which controls the amount of regularization) and the fraction of allowable boundary violations *ν*. Fig 8 shows 12 classifiers that were trained using the baseline data and different combinations of the model parameters *γ* and *ν* to illustrate their effect. An algorithm that allows no or few boundary violations (small *ν*) is said to have a hard margin. One that has more relaxed constraints on boundary violations has a soft margin. The kernel coefficient *γ* controls the width of the RBF and thereby the radius of influence of each training sample. A high *γ* limits the influence of each training sample to its close proximity and can lead to an irregular and complex decision function. When *γ* is reduced, it acts as a regularization term leading to a smoother decision function. The parameter *ν* is used to penalize boundary violations. When *ν* is small, the training algorithm ensures that a large fraction of the training samples reside within the inlier region. A relaxation of *ν* typically leads to a smaller inlier region as more training samples are allowed to fall into the outlier region. It was our training objective to accurately resolve the dynamics of the flow rate profile while keeping the decision function smooth and avoiding overfitting. Models with high regularization (top row in Fig 8) failed to represent the flow rate profile and its steep gradients, while those using a high *γ* (bottom row) resulted in an irregular decision function that would not generalize well to new data. Models with a hard margin showed a tendency to overfitting (left column), while gaps appeared in the decision function (where samples would always be classified as outliers) when *ν* was set too high (right column). The optimal set of model parameters was determined in a grid search in which various combinations of *ν* and *γ* were analyzed. The performance metric for parameter optimization was the ability of the model to discern baseline data from AR data, i.e the sensitivity of the MCI towards pathological flow. It was evaluated as the average difference between the MCI for AR and the baseline MCI. The highest sensitivity was achieved for *ν* = 0.05, and it increased with *γ*. Little gains in sensitivity could be obtained beyond *γ* = 7. Instead, the resulting models became more irregular and showed signs of overfitting, limiting their ability to generalize to new data. We tuned the model to allow for 5% training errors (*ν* = 0.05, model training score of 0.95) to account for noise in the baseline data, while avoiding gaps in the decision function. The kernel coefficient of the RBF was set to *γ* = 7 for a good compromise between sensitivity and regularization (i.e. maintaining a smooth decision function). Fig 9 illustrates the resulting baseline model that was trained on the baseline data using *ν* = 0.05 and *γ* = 7. It reflects the flow characteristics of the fully-functioning valve, including its natural variance.

**Fig 10:**
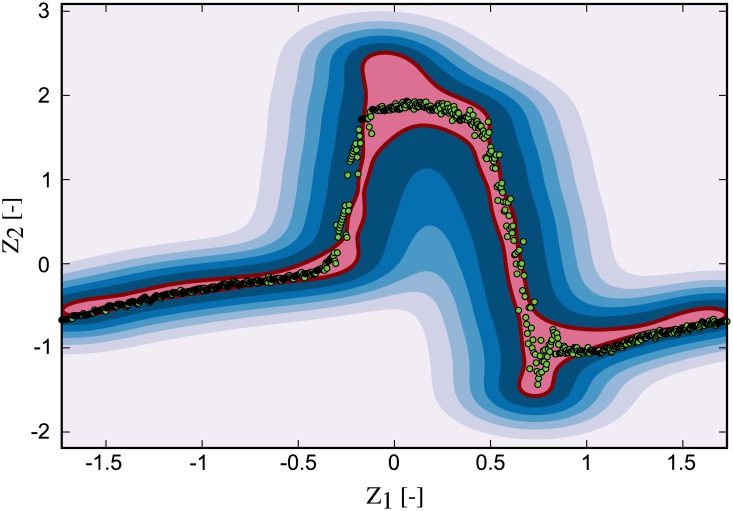
Mild AR scenario measurement overlaid onto the baseline model. Red line: decision boundary, pale red region: inlier region, shades of blue: outlier region, green dots: mild AR flow measurement.

**Fig 11:**
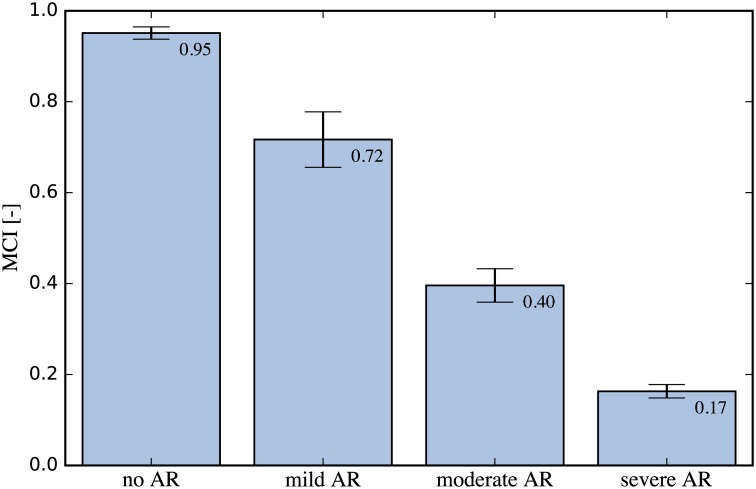
Bar plots of MCI for different AR scenarios. The MCI was computed for the baseline and for each AR scenario. A sharp decrease in MCI can be observed as artificial AR is introduced, confirming a high sensitivity of the proposed method.

For each of the three cases of AR, eight measurements with a length of 1000 samples each (∼16 cardiac cycles) were recorded with the downstream flow sensor. This data was subsequently preprocessed as outlined above and applied to the baseline SVM. The results of model prediction were then used to compute the MCI. Fig 10 shows a recording for the mild AR case, overlaid onto the baseline decision function plot. A larger fraction of the acquired samples fell outside the inlier region (pale red) when compared to the baseline measurements and these samples were thus flagged as novelty by the algorithm. This led to a decrease in MCI compared to the baseline of 0.95. This process was repeated for each of the measurements and their statistics are depicted in Fig 11 with bar plots of the MCI for the control (baseline) and each AR scenario. The blue bars indicate the median MCI and the interquartile range (iqr) is shown as error bars. All cases were significantly different, according to a one-sided Wilcoxon rank sum test at a 1% significance level. The control measurements had a median MCI of 0.95 (iqr = 0.018), equal to the model training score. Successive introduction of artificial AR led to a sharp drop in MCI. The median MCI for mild AR was 0.72 (iqr = 0.061), the median MCI of moderate AR was 0.4 (iqr = 0.037) and that of the severe AR was 0.17 (iqr = 0.015) with little agreement between measurement and baseline model.

## Discussion

The high sensitivity of the MCI with respect to AR indicates that the novelty detection algorithm is capable of identifying valvular degradation based on the flow rate recordings. Even mild AR led to a significant reduction in MCI from the baseline. Keeping the base frequency of the pulsating piston pump constant for all scenarios allowed us to reduce the problem to two features (*Z*_1_, *Z*_2_). This was done to reduce the complexity of the problem and to limit the amount of data that needed to be acquired to obtain a representative physiological baseline. However, an analogous approach may be used that includes all three features (*Z*_1_, *Z*_2_, *Z*_3_). The study of three cases of AR yielded discrete results for the MCI, which can be understood as intermediate stages of the gradual process of valvular degradation in the human body. Monitoring the MCI over time enables determination of the severity and the rate of valvular degradation which may be used to guide further action. We limited the experimental test case to the detection of AR, but the same approach may be used to detect valvular defects due to other pathologies, such as aortic stenosis. Furthermore, we limited our discussion to the detection of BHV malfunction, but the nature of this method (unsupervised, patient-specific) permits applications in other scenarios where monitoring of physiological function is of interest.

## Limitations

The in-vitro test setup, like any experimental model, is limited in its ability to accurately replicate the fluid dynamics of the human cardiovascular system. We chose boundary conditions that resemble physiological flow and pressure conditions for a human at rest. Being driven by a computer-controlled piston pump, the setup did not express, however, any significant cycle-to-cycle variability. This allowed us to tune the model for high sensitivity without generating false positives. In real-life applications, some sensitivity may have to be traded for improved robustness of the method to allow for such physiological variations. This includes the optimization of the model parameters in a physiological setting that features the full complexity of the human circulatory system. Nevertheless, the high sensitivity of the MCI with respect to valvular degradation indicates that sufficient headroom exists for higher degrees of regularization while maintaining a critical level of sensitivity to automatically detect valvular complications. In-vivo experiments, e.g. in animal trials, are required to further test the proposed methodology in an environment that is more representative of the human physiology. Comparison to classical diagnostic procedures studying the same subject could yield important insights and may reveal potential for further optimization with respect to feature selection and choice of model parameters. The use of an unsupervised learning algorithm, while providing convincing advantages, also introduces some limitations when compared to supervised classification tasks. The novelty detection algorithm is indifferent to the source of novelty and does not yield further insight into the type of underlying pathology. Thus, currently no direct link exists between the MCI and predefined medical conditions, but it rather indicates the mere presence and the evolution of a pathology. Therefore, it is meant as a monitoring tool that allows for early detection of adverse valvular behavior that can be used to initiate further medical attention.

## Future research

The limitations stated above suggest the direction of future research. This includes an animal trial to determine model parameters appropriate for in-vivo applications, and to compare our method with medical exams performed by a clinician. Of special interest will be the evolution of the MCI for progressive valve degradation and its link to diagnostic findings from echocardiography. Furthermore, the unsupervised monitoring system could be coupled with a supervised classifier to argument a detected valvular degradation with a defined pathology, such as aortic regurgitation or aortic stenosis. Such system could combine the advantages of the unsupervised and the supervised learning approach with early detection of adverse events through unsupervised anomaly detection and detailed disease diagnosis using a supervised classifier when the pathology has sufficiently manifested. Further development targets the coupling of the proposed methodology with implantable flow sensing devices to take full advantage of automation both in data processing and data acquisition. Our discussion and experimental investigations focused on the detection of heart valve diseases, but other pathologies of the cardiovascular system offer themselves to a similar approach, such as the monitoring of aortic aneurysms which may be grounds for future research.

## Conclusion

Data-driven healthcare, being fueled by the rapid development of implantable sensor technologies creates a need for automated data processing that is scalable to the high data volume of a large patient base. This automation must not be limited to preprocessing of data, but should employ the power of machine intelligence to enable computer-assisted interpretation of medical data. This is a prerequisite to enable long-term remote monitoring of patients without an excessive increase in cost. The methodology used in this article may represent an approach to tackle this challenge through the use of patient-specific models and unsupervised learning algorithms that are scalable to a large user base and adaptable to various diagnostic requirements.

## Supporting information

S1 Dataset Baseline data used to train the one-class SVM. (ZIP)

S2 Dataset Test dataset, including cases of no AR, mild AR, moderate AR and severe AR. (ZIP)
